# Prognostic significance of isochromosome 17q in hematologic malignancies

**DOI:** 10.18632/oncotarget.27914

**Published:** 2021-03-30

**Authors:** Dorota Koczkodaj, Justyna Muzyka-Kasietczuk, Sylwia Chocholska, Monika Podhorecka

**Affiliations:** ^1^Department of Cancer Genetics with the Cytogenetic Laboratory, Medical University of Lublin, Lublin, Poland; ^2^Department of Hematooncology and Bone Marrow Transplantation, Medical University of Lublin, Lublin, Poland

**Keywords:** isochromosome 17q, karyotype, FISH, hematologic malignancies, prognosis

## Abstract

Isochromosome 17q [i(17q)] with its two identical long arms is formed by duplication of the q arm and loss of the short p arm. The breakpoint in chromosome 17 that allows the formation of this isochromosome is located at 17p11.2, and the ~240 kb region with its large, palindromic, low-copy repeat sequences are present here. The region is highly unstable and susceptible to a variety of genomic alterations which may be induced by or without toxic agents. One molecular consequence of i(17q) development is the obligatory loss of a single *TP53* allele of the tumor suppressor P53 protein located at 17p13.1. Isochromosome 17q is involved in cancer development and progression. It occurs in combination with other chromosomal defects (complex cytogenetics), and rarely as a single mutation. The i(17q) rearrangement has been described as the most common chromosomal aberration in primitive neuroectodermal tumors and medulloblastomas. This isochromosome is also detected in different hematological disorders. In this article, we analyze literature data on the presence of i(17q) in proliferative disorders of the hematopoietic system in the context of its role as a prognostic factor of disease progression. The case reports are added to support the presented data. Currently, there are no indications for the use of specific treatment regimens in the subjects with a presence of the isochromosome 17q. Thus, it is of importance to continue studies on the prognostic role of this abnormality and even single cases should be reported as they may be used for further statistical analyses or meta-analyses.

## INTRODUCTION

An isochromosome is one of several structural abnormalities observed in the human genome. It is an unbalanced change in which the chromosome is comprised of two copies of either the short (p) or long (q) arm. Isochromosome 17q [i(17q)] formation is connected with concurrent duplication and partial deletion of genetic material that results in a partial trisomy of the isochromosomal arm and monosomy of the lost arm [1–3]. It has two identical long arms formed by duplication of the q arm and loss of the short p arm. The breakpoint in chromosome 17 that allows the formation of i(17q) is situated at 17p11.2, and the ~240 kb region with its large, palindromic, low-copy repeat sequences (LCR) known as “REPA” and “REPB” are located here (17p11.2) [4, 5]. The region is highly unstable and susceptible to a variety of genomic alterations which may be induced by or without toxic agents (e.g., radiation, cytostatic drugs) [5, 6]. Therefore, LCRs in the i(17q) structure account for many genetic rearrangements [4]. One molecular consequence of i(17q) development is the obligatory loss of a single *TP53* allele of the tumor suppressor P53 protein located at 17p13.1. However, studies indicate that *TP53* mutations, even at low allelic frequencies, are extremely rare in cases with i(17q). This finding may suggest that mechanisms other than *TP53* abnormality may induce genomic instability [7–10]. Thus, this complex genomic structure indicates that i(17q) occurs not as a random abnormality, but more probably due to a predisposition of the genomic structure. The molecular consequences of i(17q) presence, therefore, remain unknown [10].

Disorders of the *TP53* gene may be constitutional, as in the case of the rare Li Fraumeni syndrome and Li Fraumeni Like syndrome, while somatic mutations appear in 5% to 80% of different types of cancer depending on their type and stage [11]. In neoplasms, we observe a different frequency of *TP53* mutations, which is related to their organ localization, most often they are found in lung tumors, and least frequently in leukemias.

Mutations in the *TP53* gene and the phenomenon of loss of heterozygosity (LOH) affect the functionality of the P53 protein. The loss of the normal function of the P53 protein may result not only from point mutations in the gene, but also from a homozygous or hemizygous deletion or rearrangement in the region itself or throughout the 17p13 band.

Isochromosome 17q [(i(17q)] is involved in cancer development and progression. It is frequently detected in combination with other chromosomal defects (complex cytogenetics) and rather infrequently as a single mutation (Table 1). The i(17q) rearrangement has been described as the most common chromosomal aberration in primitive neuroectodermal tumors and medulloblastomas [5, 12]. This isochromosome is also detected in several hematological disorders such as Philadelphia positive (Ph+) chronic myeloid leukemia (CML), acute myeloid leukemia (AML), Hodgkin and non-Hodgkin lymphoma and myeloproliferative neoplasm (MPN), including MDS/MPN (myelodysplastic/myeloproliferative) overlap syndromes. Hematologic malignancies with isochromosome 17q are associated with poor prognosis and may produce distinctive clinicopathological characteristics [7, 13, 14].

**Table 1:** Gene mutations co-occurring with isochromosome 17q presence in hematologic malignancies [7, 10, 70]

Coexistent genetic abnormalities		Coexistent genetic abnormalities
**RARE**	**Izochromosome 17q**	**FREQUENT**
*TP53* *TET2*		*SETBP1* *SRSF2* *ASXL1* *NRAS*

In this article, we analyze literature data on the presence of i(17q) in proliferative disorders of the hematopoietic system in the context of its role as prognostic factor of disease progression (Table 2). The case reports are added to support the presented data.

**Table 2:** Frequency and prognostic significance of i(17q) in various hematological diseases

Disease	i(17q) frequency	i(17q) significance
Myeloproliferative/myelodysplastic neoplasms	about 1%	heterogeneity of clinical course, the precise prognostic role should be further studied [28]
Acute myeloid leukemia	about 1%	Reporter of aggressive disease and shorter survival time [8, 28]
Acute promyelocytic leukemia	0.6–4.9%	may indicate unfavourable course (due to low incidence, the precise prognostic significance should be established) [43, 52, 53]
Chronic myeloid leukemia	9–29% in patients with blast crisis	risk factor of progression to accelerated phase and blast crisis
Chronic lymphocytic leukemia	up to 4% (20–30% of all patients with a deletion of TP53)	Indicator of aggressive course and poorer prognosis [71, 72]

### Isochromosome i(17q) involvement in cancer development

The frequent presence of i(17q) in cancers such as leukemias or solid tumors, in comparison to other isochromosomes, is likely to be connected with the unique DNA sequence located at the breakpoint area of i(17q) that is observed in cancer development. This *locus* is prone to genetic rearrangement that is possibly combined with dysregulation of selective genes in the adjacent regions of the breakpoint or gene imbalances due to the loss of 17p and the increase in 17q structure [3, 5]. The mechanism of this abnormality and its molecular/genetic consequences remain inconclusive. It was proposed that the loss of a tumor suppressor gene *TP53* located on 17p13 is essential for i(17q) formation. Nevertheless, analysis of the other *TP53* allele has shown low incidence of mutations [3, 15]. The existence of the single *p53* gene may be functionally active, however its influence on other genes involved in oncogenesis can be modulated due to low expression in the presence of one copy only [5, 16, 17]. Thus, *TP53* dysfunction may result from copy number changes or alterations in RNA and protein expression of other molecules within the *TP53* pathway [10].

A study by Fioretos et al. [6] found that i(17q) in most cases of hematologic malignancies is not a monocentric, but is rather a dicentric isochromosome with two centromeres. The breakpoints of i(17q) are located, as mentioned above, within 17p11.2, either in the pericentromeric area, or within a 900-kb YAC clone located in the Smith-Magenis syndrome (SMS) common deletion region [18, 19]. This SMS is an unstable area that contains multiple low-copy repeats or segmental duplications. Abnormalities of this region are observed in the congenital SMS disorder [19, 20]. SMS is bordered by large highly homologous LCR, and forms “SMS-REPs” complex [20, 21]. SMS-REPs are substrates for nonallelic homologous recombination (NAHR) that may cause genes deletions or duplications [18, 20, 22–24]. Recently, LCRs have been shown to be mediators of NAHR responsible for numerous congenital genomic disorders and cancers [5, 7, 13].

### Isochromosome i(17q) in myeloid neoplasms

The presence of isochromosome (17q) as an isolated karyotype abnormality in myeloid malignancies is infrequent, however, it is associated with specific characteristic clinical findings [8, 25, 26]. In most cases, it occurs in myelodysplastic/myeloproliferative neoplasms (MDS/MPN), high-risk myelodysplastic syndrome, or acute myeloid leukemia (AML). It leads to distinctive morphologic characteristics such as pseudo-Pelger-Huet neutrophils and megakaryocytes of small hypolobated morphology [10, 28]. In a majority of cases, the clinical course of myeloid neoplasms with isolated i(17q) is aggressive regardless of diagnosis or blast count. Rapid progression into AML is also more common. According to the revised International Prognostic Scoring System (IPSS), the presence of isochromosome (17q) is classified as an “intermediate prognostic factor” for MDS, and as listed in the revised Medical Research Council classification, it is considered as an adverse prognostic factor for AML, which determines the use of more aggressive forms of therapy, including stem cell transplantation [8, 10, 27]. Numerous studies confirmed that i(17q) can be a prognostic marker of disease progression and shortened overall survival [28]. However, other studies report on the considerable heterogeneity of the clinical course of myeloid neoplasms with i(17q) [28].

Visconte et al. [8] described clinical characteristics and therapy outcomes of 21 patients with myeloid neoplasms with i(17)(q10), including AML, MDS and MPN, but with CML patients excluded. Typically, the patients presented leukocytosis (57%), absolute monocytosis (43%), anemia (95%), and thrombocytopenia (86%). Bone marrow examination showed dysgranulopoiesis in all cases. Moreover, the majority of patients had at least one additional gene mutation involved in granulopoiesis and leukemogenesis. In the analyzed cohort, patients with i(17)(q10) and MDS/MPN features had poor prognosis (median overall survival - OS of 4 months). However, no differences in OS were observed in the group of patients carrying i(17q) [8].

To assess clinical features and disease outcome, Ganguly et al. [28] examined patients with myeloid neoplasms and the presence of i(17q) as single or complex change. They identified 14 patients, 12 with complex and 2 with a single abnormality. In that group, the OS was 10.4 months. Of note, an increased OS was observed in the subjects who underwent hematopoietic stem cell transplantation (HSCT) in comparison to those who were on conventional chemotherapy. The authors concluded that the role of HSCT in patients with i(17q) needs further study and a larger platform of data from transplantation centers [28].

### Isochromosome i(17q) in chronic myeloid leukemia

Chronic myelogenous leukemia (CML) is a well-defined genetically myeloproliferative disorder in which the Philadelphia chromosome is present and *BCR-ABL* genes fusion is seen [29]. The disease may evolve into a blast crisis (BC) that is associated with the appearance of new genetic changes such as trisomy 8, an additional Ph chromosome, isochromosome 17q and trisomy 19 [14, 30, 31]. The involvement of i(17q) in the progression of CML has been reported, however its role in BC development remains unclear [32–34]. The incidence of i(17q) in BC is estimated at 9–29% [35–39]. Additionally, the presence of i(17q) is reported to be connected with particular hematological features, such as the increase in basophils and myeloid blast cell phenotype [40–42]. Features, like myeloid blast phenotype, blood basophilia, and the presence of i(17q) are also characteristic of an accelerated phase that frequently precedes BC [43].

### Isochromosome i(17q) in acute promyelocytic leukemia

Acute promyelocytic leukemia (APL) is a subtype of acute myeloid leukemia (AML) with the specific genetic abnormality t(15;17)(q22;q21) that results in the fusion of the retinoic acid receptor gene (*RARA*) with a gene for transcription factor (promyelocytic leukemia, or *PML*) [44–46]. Conventional t(15;17) is observed in 86–90% of all APL cases [47, 48]. Sometimes, there is no typical t(15;17) in APL, but it may be observed either in cases associated with complex chromosomal translocations between both 15, 17, and other chromosomes, or in cases with cryptic t(15;17) [49, 50]. Isochromosome 17q occurrence in APL is not high and is estimated at 0.6–4.9% [47, 48, 51, 52]. Isochromosome 17 may be involved in t(15;17) translocation and it is described as ider(17)(q10)t(15;17). In such a situation, isochromosome 17q has two copies of *RARA/PML* genes fusion on both long arms. Prognostic significance of i(17q) in APL remains controversial. Research indicates that the presence of isochromosome 17q in APL tends to follow an unfavorable course, however low incidence of this AML type makes prognostic significance rather unclear [44, 51, 52, 53].

Manola et al. [43] examined 53 patients with APL and ider(17)(q10)t(15;17). The authors concluded that i(17q) did not cause an unfavorable course of APL in the group of patients treated with ATRA (all-trans retinoic acid) and chemotherapy [43]. On the other hand, Kim et al. [51] reported on poor prognosis for ALP with ider(17)(q10)t(15;17) and in childhood APL. Beyond the aforementioned, several studies presented several cases of APL with cryptic t(15;17) on isochromosome 17 with the prognosis of short survival in most of them [52, 53]. For example, Tong et al. [52] described a case of APL with cryptic *PML/RARA* rearrangement on both long arms of isochromosome 17. The patient was treated with ATRA and chemotherapy, however the disease relapsed soon after the completion of treatment and the patient died. Thus, more studies are required to assess precisely the relationship between the presence of isochromosome and the course of APL. Currently, the presence of i(17q) in APL does not change the therapeutic approach and patients are treated with classical regimens.

### Chronic lymphocytic leukemia with isochromosome 17q

Although i(17q) is quite frequently detected in various hematological malignancies, it is rarely observed in CLL patients, and only a few studies have assessed its role in the clinical course of this type of leukemia. CLL is the most common type of adult leukemia in Western countries. It is characterized by the proliferation and accumulation of CD5 positive B lymphocytes [54, 55]. Recurrent genetic aberrations are considered the most remarkable markers of CLL prognosis [56]. Moreover, research reveals that the subjects with deletions of 17p13 and 11q22-23, as well as with *TP53*, *ATM*, *SF3B1*, *NOTCH1* mutations and unmutated *IGHV* have a poor prognosis [57–60].

The majority of subjects with del(17)(p13) have an aggressive clinical course of leukemia and belong to a high risk group [61–63]. Herein, the tumor suppressor gene *TP53* is altered by del(17)(p13). Additionally, most cases with 17p13 deletions were observed together with *TP53* mutations in the remaining allele. There is also a subgroup that carries *TP53* mutations in the absence of 17p13 deletion [64, 65]. Such alterations were observed in approximately 10% of all patients with diagnosed CLL, and were even more frequent among resistant patients with recurrent episodes (approximately 40%) [66, 67]. Del 17p13 and/or *TP53* mutations account for short survival and resistance to common chemotherapeutic regimens - with purine analogues and alkylating agents likely to cause cell death via p53-dependent apoptosis [66, 67]. However, it was reported that some patients with early stage CLL and del(17)(p13) had long survival and required no treatment [67, 68].

Genetic studies have shown that the loss of the *TP53* gene is quite frequent due to structural abnormalities in chromosome 17, such as isochromosome 17q or unbalanced translocations being even more frequent than that due to monosomy 17 or *TP53* deletions [2]. The loss of short arm and a duplication of the long arm is characteristic for i(17q). Here, the involvement of LCRs provides a mechanism for genetic rearrangements [5, 66]. Only a few studies have evaluated the clinical outcome of i(17q) in the course of CLL [69–71]. Collado et al. [70], for example, retrospectively evaluated the clinical, molecular and genetic status in 22 patients with CLL and i(17q). They described their biological characteristics, mutational *TP53* status, and *IGHV* mutations. Moreover, they analyzed the effects of such cytogenetic abnormalities with defective TP53 on leukemia outcome. The authors detected somatic *IGHV* hypermutation in all patients, and *TP53* mutations in 71.4% of all those enrolled in the study. The patients with i(17q) and complex karyotypes had poorer OS compared to the patients with other abnormalities of 17p13 [72]. Alhourani et al. [71] tried to assess if the presence of i(17q) could be a prognostic marker for CLL patients. They detected 18 cases of *TP53* deletion in a group of 150 CLL patients. Six of them had *TP53* deletion with i(17q). Other chromosomal aberrations were observed in the cases with i(17q). It may be concluded that i(17q) is a more adverse prognostic marker than *TP53* deletion alone [71].

### Case reports

This part of the on-going paper presents case reports of patients with i(17q) in the course of hematologic malignancies.

### Case report 1

**Figure 1:**
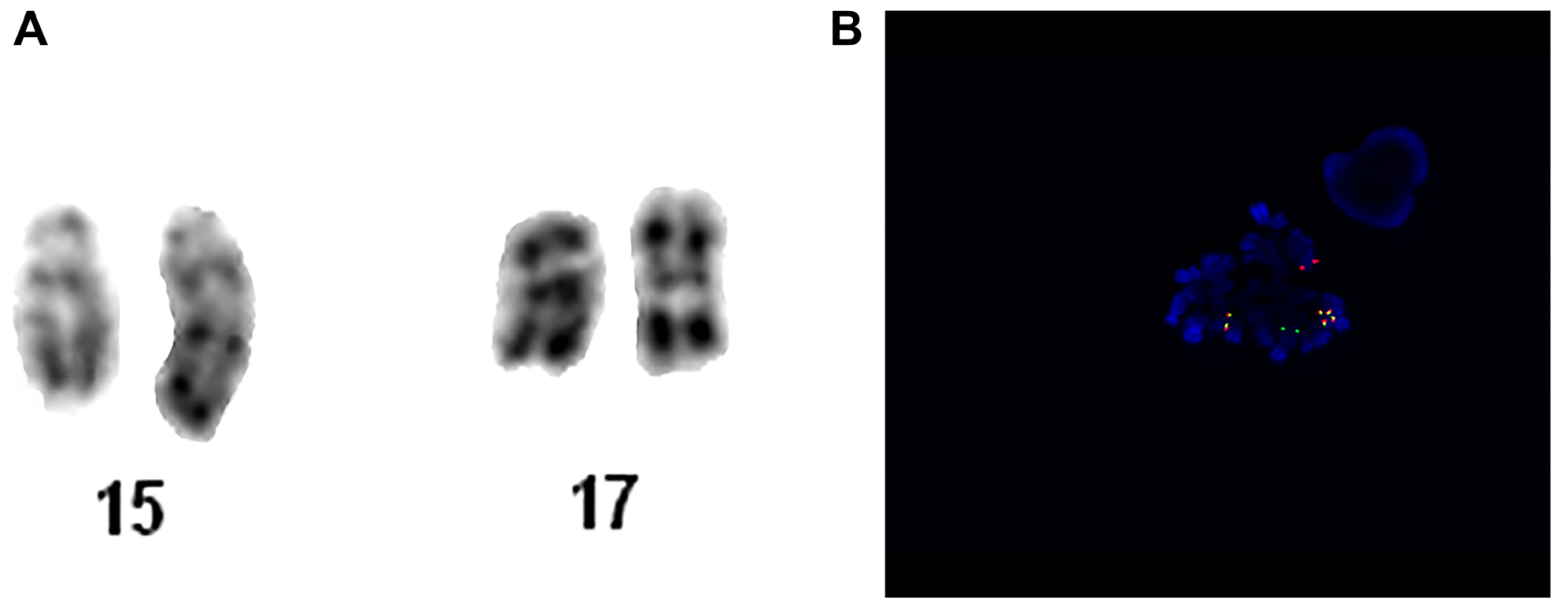
Patient no. 1. (**A**) Chromosome 15 and 17 of karyotype analysis. (**B**) Metaphase hybridized with the LSI PML/RARA Dual Color, Dual Fusion Translocation Probe. The metaphase in this image shows the one orange (PML, normal chromosome 15), one green (RARA, normal chromosome 17), one fusion (PML/RARA, der chromosome 15) and two fusion (RARA/PML, ider chromosome 17) signal pattern.

A middle-aged patient was admitted to the Hematology Department with features of cutaneous and mucosal hemorrhagic diathesis. Blood tests on admission revealed WBC at6 1.94 K/uL, Hb at 10.4 g/dL, and PLT at 47 G/L. White blood cell differential found blast cells at 2%, myelocytes at 2.9%, monocytes at 2.9%, and lymphocytes at 71.6%. Basic coagulation and biochemical tests revealed no abnormalities, except for hypofibrinogenemia with fibrinogen 1.5 g/L and slightly elevated LDH of 523 IU/L. Bone marrow biopsy confirmed the presence of 30% of all younger cells with unmatured nuclei, atrophic and atypical granules, as well as Auer’s bacilli, promyelocytes with normal granules at 28% and blast cells at 29%. Analysis of chromosomes revealed abnormal karyotype with the presence of mutual chromosomal translocation t(15;17)(q22;q21) in 4 out of 25 analyzed metaphases. FISH was performed, as addition on the short arm of chromosome 17 was suspected. An analysis of 221 interphase nuclei was undertaken and the presence of *PML/RARA* gene fusion was found in 145 cases (66%). What is more, the presence of isochromosome 17q31 in the nuclei was detected, which resulted in an additional copy of the *PML*/*RARA* gene fusion and simultaneous deletion of the *TP53*gene. Finally, the patient’s karyotype was established as: 46,XN,der(15)t(15;17)(q22;q21),ider(17)(q10)t(15;17)(q22;q21)[4]/46,XN [21]. Genetic abnormalities detected are presented in Figure 1. The final diagnosis of acute promyelocytic leukemia was confirmed by bone marrow immunophenotyping which showed the presence of app. 62% young myeloid cell lines with the immunoprotein characteristic of promyelocytes: CD34-/CD33+/CD117+/CD13+/CD64+/CD4+/CD14+/CD15+/HLA-DR-/CD65+/CD56+/MPO+.

ATRA and Idarubicin was implemented and well tolerated. The tests performed in the curse of therapy revealed complete molecular response, and subsequent consolidation cycles were prescribed. There was no side effects of treatment and complete molecular remission was achieved. At present, the patient is on maintenance therapy. Based on this case report, it can be concluded that the presence of i(17q) did not adversely affect APL treatment outcome when ATRA protocols were implemented.

### Case report 2

**Figure 2:**
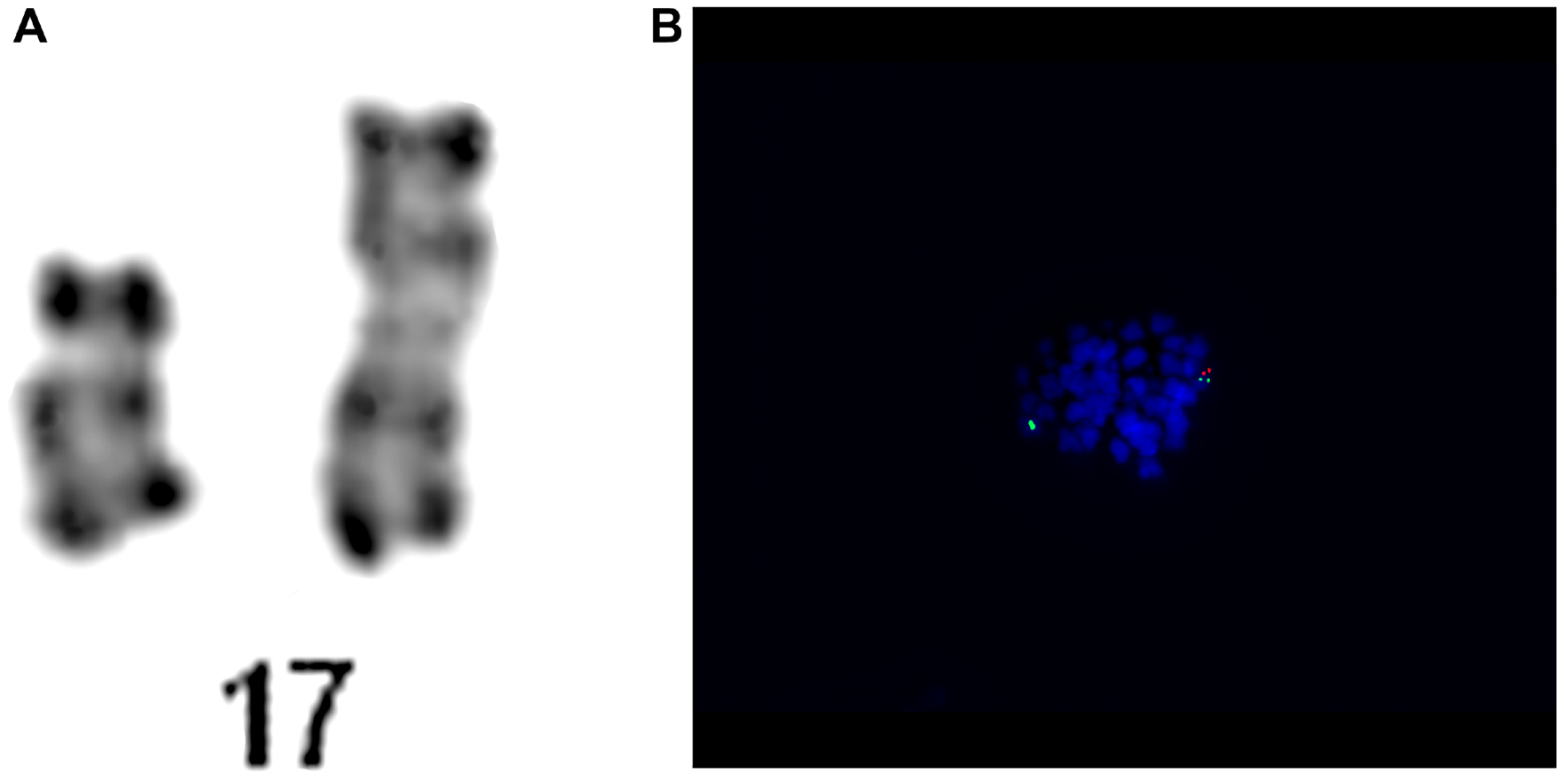
Patient no. 2. (**A**) Chromosome 15 and 17 of karyotype analysis. (**B**) Results of the hybridization of the LSI TP53/CEP17 Dual Probe Kit. The metaphase showing the two green (CEP 17) signals and one orange (TP53) signal.

A middle-aged patient was admitted to the Hematology Department presenting a weakness lasting for 2–3 weeks. Blood tests found pancytopenia with WBC at 1.97 K/uL; NEU at 0.45 K/uL; HGB at 6.6 g/dl; PLT at 48 K/uL. Supportive transfusions of red blood cells and platelets were required. Bone marrow was biopsied to search for an underlying cause. It revealed medium-cellular bone marrow with normoblastic renewal, features of dysplasia in the red blood cell system, reduced granulocytes, impaired ability to form platelets, and blasts cells at 17%. Based on the bone marrow cell immunophenotyping, the following phenotypes were found: CD34+; CD117+; CD33low; DR+; CD13+; CD11c-; CD15-; CD64-; CD2-; CD7-; CD19; CD65-; CD14-; CD4; MPO-. Myelodysplastic syndrome (MDS RAEB 2) was diagnosed. Molecular studies found no tandem duplication or D835 in *FLT3* gene, and no mutation in *NPM1* gene. However, a change was found in the heterozygous substitution system in *CEBPA* gene. Karyotyping revealed numerous abnormalities: 46~50,XN,-5,+8,-16,i(17)(q10),-18,-19,+add(20)(q13),+del(22)(q11)x2,+1~2mar[cp27]/46,XN. Analysis of chromosomes with GTG and RHG staining showed a complex karyotype with monosomy of chromosomes 5, 16, 18, 19 in 27 metaphases, and trisomy of 8-pair chromosome. Furthermore, structural changes such as isochromosome on the long arm of chromosome 17 [i(17)(q10)] and addition on the long arm of chromosome 20 [add(20)(q13)] were observed, together with increased copies of this chromosome. Moreover, additional chromosome 22 deletions of the long arm fragment [del(22)(q11)] and marker chromosomes were also detected. The correct karyotype was found only in 3 metaphases. The patient was enrolled into the high cytogenetic risk group. Genetic abnormalities detected are presented in Figure 2.

Initially, the patient was treated with azacytidine. In the course of therapy, a control bone marrow biopsy was performed. It revealed 27% blasts cells with the following phenotype: CD34+, CD117+, CD4low, CD33+, CD14-, CD64-, CD65-, CD56-, DR+, CD13-+ (7%), CD11c-+ (9%), MPO-. The result suggested MDS transformation into AML and intensive therapy was required. The patient was prescribed chemotherapy with daunorubicin, cladribine, and cytarabine. The subsequent hematological tests confirmed complete remission, however, with incomplete regeneration of the thrombotic system. Thus, consolidating chemotherapy was continued. Due to high risk, the patient was qualified for bone marrow transplantation from an unrelated donor. After myeloablative Flu-TBI (total body irradiation) -ATG conditioning, the patient received allotransplantation of hematopoietic cells from the peripheral blood from an unrelated donor (alloURD HCT). At present, the patient’s condition is good, and no active infection, no symptoms of aGVHD, no organ changes indicating PTLD (post-transplant lymphoproliferative disorders) or other post-transplant complications have been observed. Laboratory tests found a stable hematopoietic system without lymphoproliferative features, and normal liver and kidney function. Based on the presented case, it can be concluded that intensive treatment with bone marrow transplantation is an effective therapeutic option, despite high risk genetic changes.

### Case report 3

**Figure 3:**
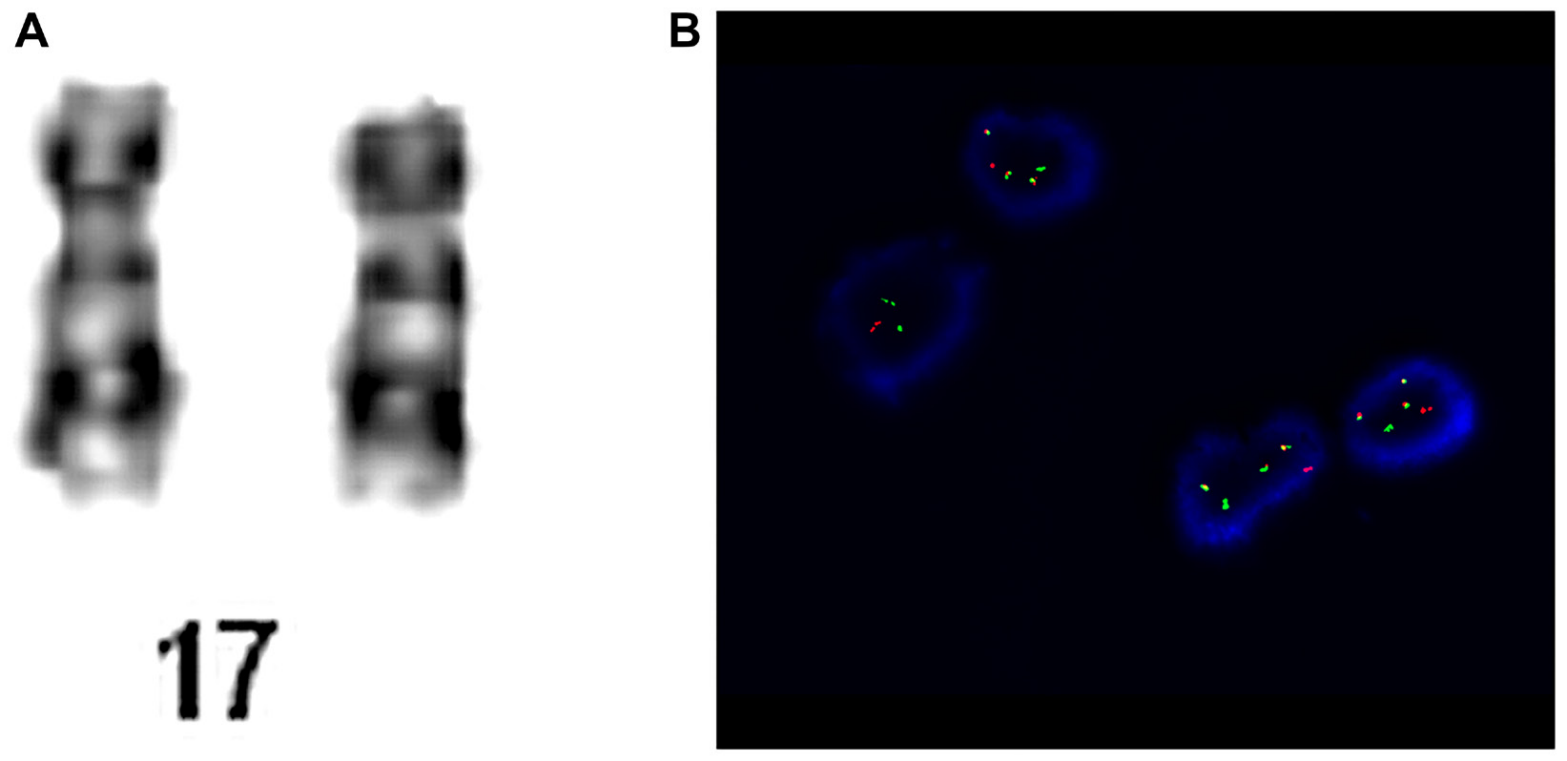
Patient no. 3. (**A**) Chromosome 17 of karyotype analysis. (**B**) Cells hybridized with the LSI PML/RARA Dual Color, Dual Fusion Translocation Probe. The cells in this image show the one orange (PML, normal 17 chromosome), one green (RARA, normal 17 chromosome), one fusion (PML/RARA, der 15 chromosome) and two fusion (RARA/PML, ider 17 chromosome) signal pattern.

A young adult patient was admitted to the Hematology Department presenting anemia, leukopenia and deep thrombocytopenia. The patient reported weakness and significantly worsened general condition for about 2 weeks. The patient was on antibiotics for urinary tract infections. On admission, the patient was in generally good condition, without features of cardiopulmonary insufficiency. WBC revealed normocytic anemia with HGB concentration at 7.4 g/dL and MCV at 86.6 fL, leukopenia with neutropenia (WBC at 1.76 K/uL and NEU at 0.26 K/uL, respectively), and deep thrombocytopenia with PLT at 6 K/uL. Peripheral blood smear revealed the presence of promyelocytes –1%, myelocytes –1%, metamyelocytes –1%, segments –8%, eosinophils –2%, basophils –4%, lymphocytes –61%, monocytes –22%. Basic hemostasis tests found no abnormalities, except for a significantly increased D-dimer concentration up to 20043 ng/ml. Biochemical tests revealed slightly elevated LDH at 526 IU/L, and an increase in GGTP to 200 Iu/L. Bone marrow biopsy with immunophenotype and genetic tests was performed. Chromosomal analysis revealed normal karyotype 46, XN, however, FISH detected the presence of i(17)(q10). The cytogenetic analysis are presented in Figure 3. The diagnosis of acute promyelocytic leukemia was established and the patient was qualified for the treatment protocol with ATRA and idarubicin. Due to anemia and thrombocytopenia, the patient received RBC and platelet transfusion. However, before the onset of treatment, the patient’s condition worsened, he was confused and sleepy. CT scan of the head showed hypodense areas in the entire temporal lobe of the left hemisphere indicating intracerebral bleeding. The patient’s condition deteriorated progressively. Massive gastrointestinal and respiratory bleeding was observed despite supplementation therapy, and the patient died on the next day. Based on this case report, no prognostic significance of i(17q) in the treatment efficacy or survival time could be determined. However, the presence of this genetic change could have caused a more progressive course of leukemia with very deep cytopenia that finally led to the patient’s death.

## CONCLUSIONS

Since i(17q) is the most commonly observed isochromosome in hematological neoplasms and solid tumors, it is worth to conduct research evaluating its role in the course of these cancers. According to the Mitelman Database of Chromosome Aberrations in Cancer (http://cgap.nci.nih.gov/Chromosomes/Mitelman), i(17q) is observed in approximately 2.5% of all cases [70]. The results published to date have indicated the involvement of i(17q) in both myeloproliferative and lymphoproliferative disorders, however, the number of cases presented has been rather small. Moreover, i(17q) is detected either as a single abnormality or as a part of a complex karyotype, which additionally influences its prognostic significance and makes the results of research unclear. Thus, it is of importance to continue studies on the role of i(17q) in both aggressive and indolent hematologic malignancies. In addition, i(17q) needs to be investigated as a single occurrence and as a part of complex karyotype in bigger groups. Even single cases should be reported as they may be used for further statistical analyses or meta-analyses.
