# Addendum: Prognostic significance of isochromosome 17q in hematologic malignancies

**DOI:** 10.18632/oncotarget.28295

**Published:** 2023-02-11

**Authors:** Dorota Koczkodaj, Justyna Muzyka-Kasietczuk, Sylwia Chocholska, Monika Podhorecka

**Affiliations:** ^1^Department of Cancer Genetics with the Cytogenetic Laboratory, Medical University of Lublin, Lublin, Poland; ^2^Department of Hematooncology and Bone Marrow Transplantation, Medical University of Lublin, Lublin, Poland

**Consent was added to this article:** Patient consent was required and obtained. The authors received approval from the Medical University Ethics committee to perform the genetic studies presented.

Original article: Oncotarget. 2021; 12:708–718. 708-718. https://doi.org/10.18632/oncotarget.27914

